# The Prognostic Role of NEDD9 and P38 Protein Expression Levels in Urinary Bladder Transitional Cell Carcinoma

**DOI:** 10.1155/2017/6095205

**Published:** 2017-01-17

**Authors:** Ola A. Harb, Rasha Haggag, Maged M. Ali, Shereen El Shorbagy, Abeer M. Abdelbary, Lobna A. Abdelaziz, Reham A. Salim, Khaled M. Abdel Wahab

**Affiliations:** ^1^Department of Pathology, Faculty of Medicine, Zagazig University, Sharkia, Egypt; ^2^Department of Medical Oncology, Faculty of Medicine, Zagazig University, Sharkia, Egypt; ^3^Department of Urology, Faculty of Medicine, Zagazig University, Sharkia, Egypt; ^4^Department of Clinical Oncology and Nuclear Medicine, Faculty of Medicine, Zagazig University, Sharkia, Egypt

## Abstract

*Background.* The most common malignant tumor of the urinary bladder is transitional cell carcinoma (TCC). Neural precursor cell-expressed developmentally downregulated protein 9 (NEDD9) is found to be a cell adhesion mediator. P38 Mitogen-Activated Protein Kinase is a serine/threonine kinases member which can mediate carcinogenesis through intracellular signaling.* Methods.* To assess their prognostic role; NEDD9 and p38 protein were evaluated in sections from 50 paraffin blocks of TCC.* Results.* The high expressions of NEDD9 and p38 protein were significantly associated with grade, stage, distant metastasis (*p* < 0.001), number of tumors, lymph node metastasis, and tumor size (*p* < 0.001, 0.002; 0.018, <0.001; and 0.004, 0.007, respectively). High NEDD9 and p38 detection had a worse 3-year OS (*p* = 0.041 and <0.001, respectively). By multivariate analysis the NEDD9 and p38 protein expression levels and various clinicopathological criteria including gender, grade, stage of the tumor, and regional lymph node involvement were independent prognostic parameters of TCC of the urinary bladder patients' outcome.* Conclusion.* NEDD9 and p38 protein expressions were poor prognostic markers of TCC.

## 1. Introduction

Bladder cancer is the 4th commonest malignancy worldwide and the 8th cause of cancer related mortality among males with men to women ratio of 3 : 1. In the United States, an estimated 74,690 new cases of bladder cancer and 15,580 deaths occurred in year 2014 [1].

In Egypt, bladder malignancies were the commonest among urinary system malignant tumors (90.71%) and the third among all malignancies [2]. Most of patients with bladder carcinoma had 30%–70% recurrence rate and 10%–30% of them may progress to muscle-invasive tumors [3]. Transitional cell carcinoma (TCC) forms more than 90% of all cases of malignancies of the urinary bladder [4], and detection of biomarkers, molecular mechanisms, and new immunohistochemical markers are helpful in expecting its evolution. NEDD9 (neural precursor cell-expressed developmentally downregulated protein 9) also known as HEF1 (human enhancer of filamentation 1) is a cytoskeletal protein which is considered a signaling mediator of various cellular events including cell adhesion, cell cycle regulation, apoptosis, and tumorigenesis [5].

p38 MAPK (Mitogen-Activated Protein Kinase) is a class of serine/threonine kinases that mediate intracellular signaling associated with a variety of cellular activities including cell proliferation, differentiation, survival, death, and transformation [6].

As previous studies implicated that NEDD9 and p38 protein have a promoting effect in the carcinogenic process in many other organs, however, the prognostic value of studying the expression of both of them in urinary bladder transitional cell carcinoma has not been investigated yet, so we have chosen such markers.

In this study we aimed to assess the expression of NEDD9 and p38 protein and detect their prognostic role in urinary bladder TCC.

## 2. Patients and Methods

This retrospective cohort study was conducted at Pathology, Urology, and Medical Oncology and Clinical Oncology and Nuclear Medicine Departments, Faculty of Medicine, Zagazig University. Archival formalin fixed paraffin-embedded 50 tissue blocks derived from 50 cases each block taken from one patient of transitional cell carcinoma of the urinary bladder were collected from patients previously undergone transurethral bladder resection (TUR) between November 2012 and November 2015.

This study complied with the guidelines of the local ethics committee and was approved by the Zagazig University IRB.

The TNM staging system was used for pathologic staging [7], and the World Health Organization classification was used for pathologic grading of bladder TCC [8]. Each tumor was reevaluated by retrospective examination of the medical records and the slide file of the Pathology Department, sex, age, tumor size, histological subtype, grade, depth of muscle invasion, status of lymph node metastasis, and distant metastasis.

### 2.1. Immunohistochemical Staining

Immunohistochemical staining was carried out using the streptavidin-biotin immunoperoxidase technique [9]. 4 *μ*m thick sections were cut from formalin fixed paraffin-embedded blocks, put on positively charged slides, deparaffinized in xylene, and rehydrated in graded alcohol. Sections were boiled in citrate buffer (pH 6.0) for 20 min and then washed in phosphate buffer saline (pH 7.3).

We have used 3% hydrogen peroxide in methanol to overcome endogenous peroxidase activity, followed by incubation with 1% bovine serum albumin to reduce background nonspecific binding.

Then the slides were incubated overnight with mouse monoclonal anti-NEDD9 antibody (NEDD9 clone ab18056, dilution 1 : 100 Abcam, Cambridge, UK) and rabbit polyclonal anti-p38 protein antibody (clone ab197348) dilution 1 : 100 Abcam, Cambridge, UK, at 41C in a humid chamber. A biotin-labeled secondary antibody was added for 15 min, followed by horseradish peroxidase for 15 min. Tissues were then stained for 5 min with diaminobenzidine tetrahydrochloride (DAB) and counterstained with hematoxylin.

Pancreatic adenocarcinoma was used as positive control for NEDD9 [10] while colon adenocarcinoma was used for p38 protein. Negative controls were performed by omitting of the primary antibody and replacement with phosphate buffered saline.

### 2.2. Evaluation of Immunostaining of Both Markers in TCC Cells

NEDD9 was cytoplasmic, nonspecific membranous staining and may be detected but we depend in our scoring only on cytoplasmic immunoexpression, and P38 was nuclear.

We have reviewed and scored the degree of immunostaining based on the intensity of staining and the percentage of immunoreactive cells.

We defined low and high expression by the following steps:

1. Staining intensity was graded according to the following criteria: 0 (no staining); 1 (weak staining = light yellow); 2 (moderate staining = yellowish brown); and 3 (strong staining = brown).
2. The extent of immune-reactivity was graded as follows: 0, positive cells less than 1%; (1) 2%–25%; (2) 26%–50%; (3) 51%–75%; (4) more than 75%.
3. The total score was calculated by multiplying the intensity and extent, and the samples were divided into four categories according to the following grades: 0-1 (−); 2–4 (+); 5–8 (++); and 9–12 (+++).
4. An optimal cut-off value was identified as follows: a staining index score of more than 4 was used to define tumors with high HEF1 expression, and a staining index score less than/or equal to 4 was used to indicate low HEF1 expression.

**Figure 1:**
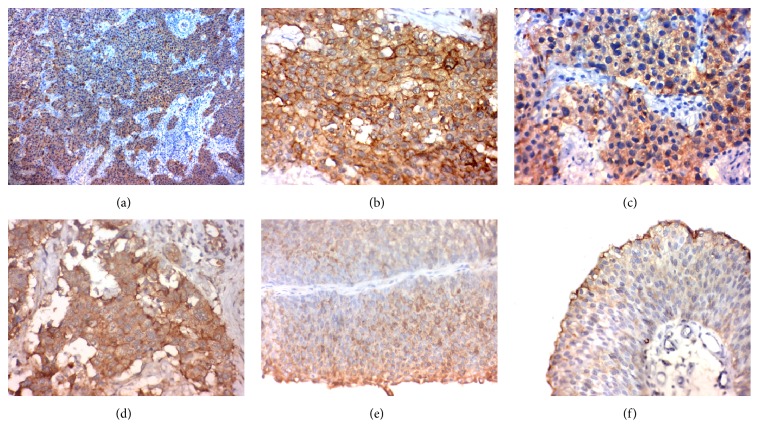
Immunohistochemical staining of NEDD9 in transitional cell carcinoma.* Notes*. (a)–(d) High NEDD9 immunohistochemical expression (in the cytoplasm) in muscle-invasive high grade transitional cell carcinoma of the urinary bladder (↑). (e)-(f) Low NEDD9 immunohistochemical expression in non-muscle-invasive low grade transitional cell carcinoma of the urinary bladder a (↑). Magnification: (a) the original magnification was ×100 and (b)–(f) the original magnification was ×400. NEDD9, neural precursor cell-expressed developmentally downregulated protein 9; TCC, transitional cell carcinoma.

**Figure 2:**
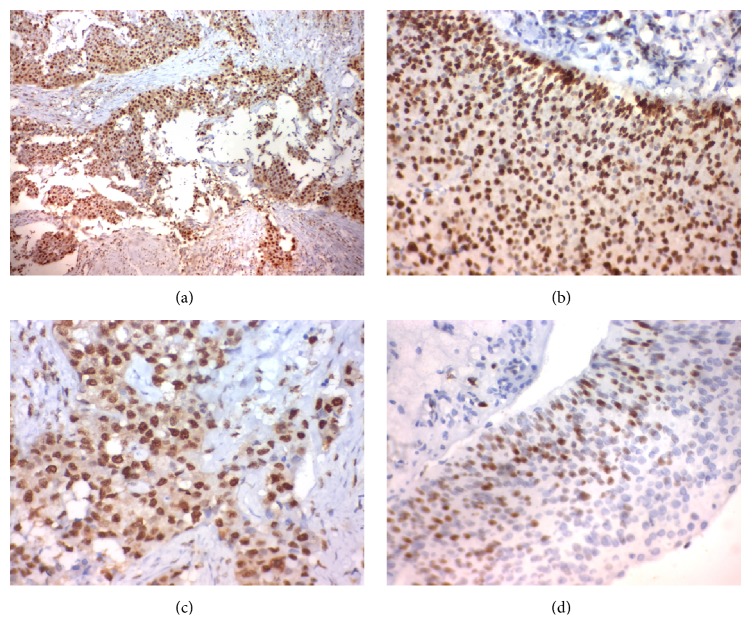
Immunohistochemical staining of P38 in transitional cell carcinoma.* Notes*. (a)-(b) High P38 immunohistochemical expression (in the nucleus) in muscle-invasive high grade transitional cell carcinoma of the urinary bladder (↑). Magnification: the original magnification was ×400. (c)-(d) Low P38 immunohistochemical expression in non-muscle-invasive low grade transitional cell carcinoma of the urinary bladder (↑). Magnification: (a) the original magnification was ×100. (b)–(d) The original magnification was ×400.

The expression of both of our markers was uniform across the tissue section and we calculate the intensity and extent of stain semiquantitatively in the maximum areas of expression ([11, 12]; Figures 1 and 2).

### 2.3. Statistical Analysis

All statistics were performed using SPSS 22.0 for windows (SPSS Inc., Chicago, IL, USA) and MedCalc windows (MedCalc Software bvba 13, Ostend, Belgium). Strength of relationship between NEDD9, p38, and clinicopathological features was determined by computing appropriate correlation coefficient (Spearman's). Kaplan-Meier method was used to estimate survival curves and comparison between groups was done by long-rank test. A *p* value <0.05 was considered statistically significant. Multivariate analyses were done using the Cox proportional hazards regression model (Table 5).

## 3. Results

Fifty patients, 35 males and 15 females, were enrolled in this study, with age ranging from 35 to 75 years (mean ± SD: 55.08 ±  10.36), Table 1.

NEDD9 expression and its correlation clinicopathological features are as follows:

(i) The expression of HEF1 in TCC of the bladder was significantly associated with number of tumors, depth of muscle invasion, grade and stage, and distant metastasis (*p* < 0.001), lymph node metastasis (*p* = 0.018), and tumor size (*p* = 0.004), but it had no association with age (Table 1; Figure 1).

P38 protein expression and its correlation clinicopathological features are as follows:

(i) The expression of p38 MAPK in TCC of the bladder was significantly associated with depth of muscle invasion, grade, stage, lymph node metastasis, distant metastasis (*p* < 0.001), size (*p* = 0.007), and number of tumors (*p* = 0.002), but it had no association with age (Table 1: Figure 2).
(ii) The expression of both NEDD9 and p38 was significantly positively correlated with each other *r* = +0.460 (*p* < 0.001).
(iii) The sensitivity of combination of both NEDD9 and p38 as a predictor for deep muscle invasion of TCC of the bladder was 73.1% and the specificity is 100%.

*Survival Analysis*. The 3-year OS rate was 52% for all patients, 44.1% and 82.4% for high and low NEDD9 expression, respectively, and 20.5% and 82.4% in high and low p38 protein expression, respectively, and the 3-year OS rate is inversely related to high NEDD9 immunoreactivity and high P38 protein expression (*p* = 0.041 and <0.001, resp.; Table 2).

**Figure 3:**
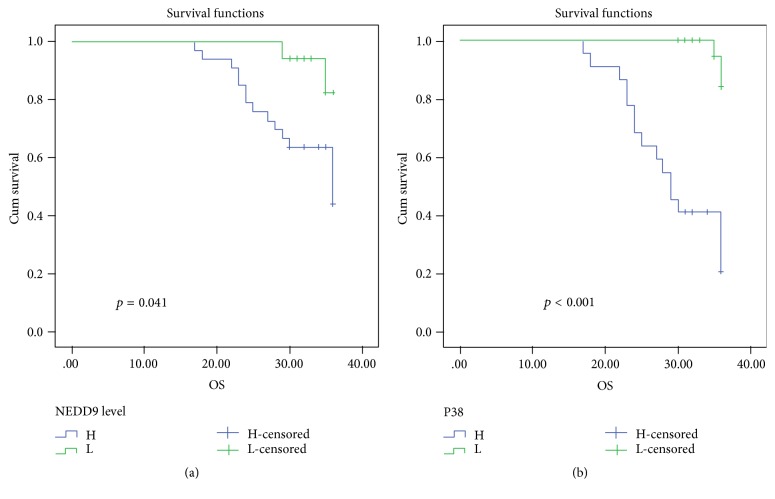
Kaplan-Meier plot of overall survival: (a) stratified according to NEDD9 and (b) stratified according to P38 protein expression.

**Figure 4:**
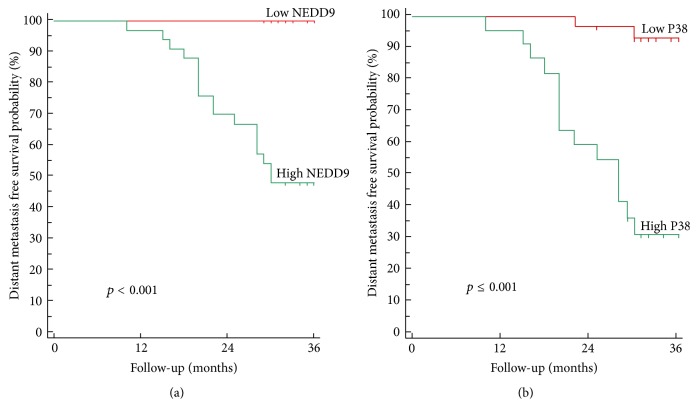
Kaplan-Meier plot of disease free survival: (a) stratified according to NEDD9 and (b) stratified according to P38 protein expression.

The 3-year DFS rate was 65.6% for all patients, 47.6% and 100% in high and low NEDD9 expression, respectively, and 31.2% and 92.7% in high and low p38 protein expression, respectively, and highly significant inverse relationship was found between 3-year DFS and both high NEDD9 protein expression and high p38 protein expression (*p* < 0.001; Table 3; Figures 3 and 4).

**Figure 5:**
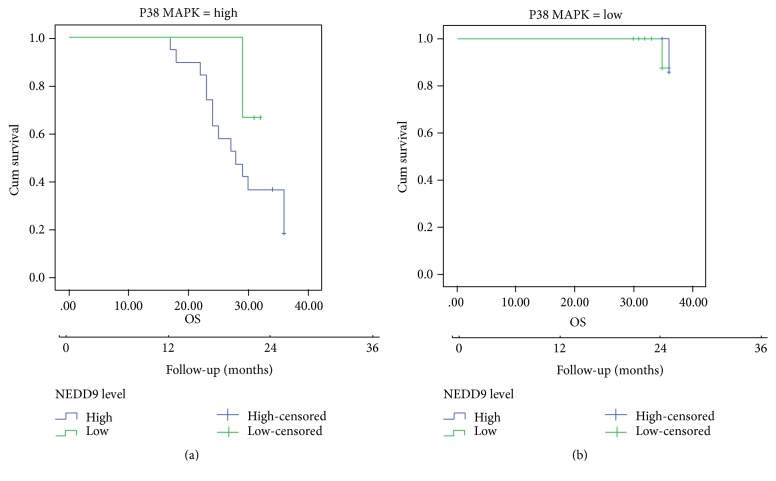
Overall survival Kaplan-Meier plot using both p38 MAPK and NEDD9 together.

Overall survival Kaplan-Meier plot using both p38 MAPK and NEDD9 together to see if better patient stratification is possible showed that patients with high p38 MAPK expression had mean OS of 28.526 ± 1.538 versus 31 ± 0.816 months in patients with high and low NEDD9 protein expression, respectively, while patients with low p38 MAPK expression had mean OS of 35.875 ± 0.0 versus 35.944 ± 0.11 months in patients with high and low NEDD9 protein expression, respectively (*p* = 0.46; Figure 5).

By multivariate analysis NEDD9 and p38 protein expression levels and various clinicopathological features (gender, stage, grade, number of tumors, and regional lymph node involvement) were independent prognostic parameters of bladder cancer patients' outcome (Table 4).

## 4. Discussion

We found that the detection of NEDD9 in TCC of the bladder was significantly positively correlated with number of tumors, invasion depth, grade and stage, distant metastasis (*p* < 0.001), lymph node metastasis (*p* = 0.018), and tumor size (*p* = 0.004). Regarding outcome of our patients we found that the detection of NEDD9 in TCC of the bladder was significantly positively correlated with shortened progression-free survival (OS and DMFS) rates (*p* = 0.041). Our results indicate that NEDD9 is involved in the growth and progression of TCC and it may be an important biological marker for its invasion and metastasis. That was consistent with results of previous studies about TCC of the urinary bladder [13] and cancers of other organs such as stomach [14], lung [15], glioblastoma [16], melanoma, [17], and breast [18]. We found that NEDD9 expression is related to an aggressive behavior in TCC. Liu et al., 2014, proved that. That was explained by several mechanisms as it supports the activation of oncogenic signaling pathways in breast cancer development. In colorectal malignancy, NEDD9, a novel target of Wnt signaling, and its high expression increased colonic cell invasion [19].

We proved that the expression of p38 protein in TCC of the bladder was significantly positively related to poor prognosis and progression and was significantly associated with shortened progression-free survival (OS and DMFS), *p* < 0.001. That was similar to results of previous studies as NEDD9 regulates MMP-2 and MMP-9 expression in TCC of the bladder cells that increase its invasion and metastases [20]. Our results were similar to the results of Thomas et al., 2014, who proved that also activation of p38 protein is associated with disease progression in head and neck squamous cell carcinoma [21]. Our findings were in agreement with researches that found that increased levels of activated p38 protein have been correlated with aggressive behavior of leukemia, breast, prostate, gastric cancers, and follicular lymphoma [22]. Activation of Cas-family proteins had been incriminated as signaling molecules in cellular motility, apoptosis, and oncogenic transformation. HEF1/Cas-L (NEDD9) is a Cas-family member whose production is important to increase levels of mRNA transcripts that encode proteins that are associated with motility, cell conversion, and invasiveness. Upregulation of such proteins suggests mechanisms through which misregulation of NEDD9 may be involved in cancer progression. Our work has proved the axis that links Cas-family protein (NEDD9/Cas-L) to the activation of p38 protein signaling pathway and that was in agreement Law et al., who found that NEDD9 overproduction results in activation of p38 protein, as NEDD9 rapidly induces changes in cellular morphology and motility, enhancing cell speed and haptotaxis towards fibronectin in a process partially dependent on intact p38 protein signaling pathway [23]. Law et al., 2000, stated that p38 kinase is activated by NEDD9 [23] and that activation of p38 kinase has also been implicated in cellular mobility [24].

The previous studies explained our finding of a significant positive correlation between the expression of NEDD9 and p38 protein (*p* = 0.007), indicating that there are synergistic effects between them during the development of invasion and metastases of transitional cell carcinoma of the urinary bladder; also their pattern and intensity of expression together can help to detect the prognosis of transitional cell carcinoma and predict patient outcome and survival.

## 5. Conclusion

NEDD9 and p38 protein may represent new prognostic molecular markers of TCC of the urinary bladder in relation to other markers.

## Figures and Tables

**Table 1:** Clinicopathological features, immunohistochemical markers, and outcome of 50 patients with TCC of bladder.

Characteristics	All		NEDD9				*p* value	P38				*p* value
			Low (*N* = 17)		High (*N* = 33)			Low (*N* = 28)		High (*N* = 22)		
	(*N* = 50)											
	Number	(%)	Number	(%)	Number	(%)		Number	(%)	Number	(%)	
Age (years)												
Mean ± SD	55.08 ± 10.36		55.2 ± 12.37		54.96 ± 9.37		0.766^‡^	51.75 ± 10.26		59.31 ± 9.03		0.013^‡^
Median (range)	52	(35–75)	56	(35–72)	50	(43–75)		49	(35–72)	61	(43–75)	
≤60 years	32	(64%)	10	(58.8%)	22	(66.7%)	0.584^§^	21	(75%)	11	(50%)	0.068^§^
>60 years	18	(36%)	7	(41.2%)	11	(33.3%)		7	(25%)	11	(50%)	

Sex												
Male	35	(70%)	5	(29.4%)	30	(90.9%)	<0.001^§^	13	(46.4%)	22	(100%)	<0.001^§^
Female	15	(30%)	12	(70.6%)	3	(9.1%)		15	(53.6%)	0	(0%)	

Size (cm)												
Mean ± SD	3.74	±2.74	2.35	±1.49	4.45	±2.0	0.015^‡^	2.89	±2.42	4.81	±2.80	0.010^‡^
Median (range)	2	(1–10)	2	(1–6)	2.98	(1–10)		2	(1–10)	6	(1–9)	
≤5 cm	34	(68%)	16	(94.1%)	18	(54.5%)	0.004^§^	24	(85.7%)	10	(45.5%)	0.002^§^
>5 cm	16	(32%)	1	(5.9%)	15	(45.5%)		4	(14.3%)	12	(54.5%)	

Multiplicity												
Absent	17	(34%)	17	(100%)	0	(0%)	<0.001^§^	14	(50%)	3	(13.6%)	0.007^§^
Present	33	(66%)	0	(0%)	33	(100%)		14	(50%)	19	(86.4%)	

Grade												
Low grade	13	(26%)	13	(76.5%)	0	(0%)	<0.001^§^	13	(46.4%)	0	(0%)	<0.001^§^
High grade	37	(74%)	4	(23.5%)	33	(100%)		15	(53.6%)	22	(100%)	

Deep invasion												
Absent	24	(48%)	16	(94.1%)	8	(24.2%)	<0.001^§^	22	(78.6%)	2	(9.1%)	<0.001^§^
Present	26	(52%)	1	(5.9%)	25	(75.8%)		6	(21.4%)	20	(90.9%)	

T												
Ta	5	(10%)	5	(29.4%)	0	(0%)	<0.001^*∗*^	5	(17.9%)	0	(0%)	<0.001^*∗*^
T1	19	(38%)	11	(64.7%)	8	(24.2%)		17	(60.7%)	2	(9.1%)	
T2	22	(44%)	1	(5.9%)	21	(63.6%)		6	(21.4%)	16	(72.7%)	
T3	4	(8%)	0	(0%)	4	(12.1%)		0	(0%)	4	(18.2%)	

Lymph node												
Negative	36	(72%)	16	(94.1%)	20	(60.6%)	0.018^§^	27	(96.4%)	9	(40.9%)	<0.001^§^
Positive	14	(28%)	1	(5.9%)	13	(39.4%)		1	(3.6%)	13	(59.1%)	

NEDD9												
Mean ± SD	34.54 ± 24.15		8.64 ± 4.83		47.87 ± 18.49		<0.001^‡^	26.85 ± 24.43		44.31 ± 20.37		0.005^‡^
Median (range)	30	(3–90)	7	(3–20)	50	(20–90)		20	(3–90)	50	(5–80)	
Low	17	(34%)						14	(50%)	3	(13.6%)	0.007^§^
High	33	(66%)						14	(50%)	19	(86.4%)	

P38												
Mean ± SD	44.1 ± 28.17		30.29 ± 24.71		51.21 ± 27.50		0.016^‡^	20.53 ± 7.24		74.09 ± 10.07		<0.001^‡^
Median (range)	30	(10–90)	20	(10–90)	60	(10–90)		20	(10–35)	70	(60–90)	
Low	28	(56%)	14	(82.4%)	14	(42.4%)	0.007^§^					
High	22	(44%)	3	(17.6%)	19	(57.6%)						

Categorical variables were expressed as number (percentage); continuous variables were expressed as mean ± SD and median (range).

^‡^Mann–Whitney *U* test; ^§^Chi-square test; ^*∗*^Chi-square test for trend; *p* < 0.05 is significant.

**Table 2:** Relation between clinicopathological features and immunohistochemical markers in the 50 patients with TCC of bladder.

Characteristics	All		NEDD9				*p*	P38				*p*
			Low (*N* = 17)		High (*N* = 33)			Low (*N* = 28)		High (*N* = 22)		
	(*N* = 50)											
	Number	(%)	Number	(%)	Number	(%)		Number	(%)	Number	(%)	
Age (years)												
Mean ± SD	55.08 ± 10.3		55.29 ± 12.3		54.96	±9.37	0.766^‡^	51.7 ± 10.265		59.3 ± 9.031		0.013^‡^
Median (range)	52	(35–75)	56	(35–72)	50	(43–75)		49	(35–72)	61	(43–75)	
≤60 years	32	(64%)	10	(58.8%)	22	(66.7%)	0.584^§^	21	(75%)	11	(50%)	0.068^§^
>60 years	18	(36%)	7	(41.2%)	11	(33.3%)		7	(25%)	11	(50%)	

Sex												
Male	35	(70%)	5	(29.4%)	30	(90.9%)	<0.001^§^	13	(46.4%)	22	(100%)	<0.001^§^
Female	15	(30%)	12	(70.6%)	3	(9.1%)		15	(53.6%)	0	(0%)	

Size (cm)												
Mean ± SD	3.74 ± 2.7		2.35 ± 1.4		4.4 ± 2.05		0.015^‡^	2.89	±2.42	4.8 ± 2.8		0.010^‡^
Median (range)	2	(1–10)	2	(1–6)	2.9	(1–10)		2	(1–10)	6	(1–9)	
≤5 cm	34	(68%)	16	(94.1%)	18	(54.5%)	0.004^§^	24	(85.7%)	10	(45.5%)	0.002^§^
>5 cm	16	(32%)	1	(5.9%)	15	(45.5%)		4	(14.3%)	12	(54.5%)	

Multiplicity												
Absent	17	(34%)	17	(100%)	0	(0%)	<0.001^§^	14	(50%)	3	(13.6%)	0.007^§^
Present	33	(66%)	0	(0%)	33	(100%)		14	(50%)	19	(86.4%)	

Grade												
Low grade	13	(26%)	13	(76.5%)	0	(0%)	<0.001^§^	13	(46.4%)	0	(0%)	<0.001^§^
High grade	37	(74%)	4	(23.5%)	33	(100%)		15	(53.6%)	22	(100%)	

Deep invasion												
Absent	24	(48%)	16	(94.1%)	8	(24.2%)	<0.001^§^	22	(78.6%)	2	(9.1%)	<0.001^§^
Present	26	(52%)	1	(5.9%)	25	(75.8%)		6	(21.4%)	20	(90.9%)	

T												
Ta	5	(10%)	5	(29.4%)	0	(0%)	<0.001^*∗*^	5	(17.9%)	0	(0%)	<0.001^*∗*^
T1	19	(38%)	11	(64.7%)	8	(24.2%)		17	(60.7%)	2	(9.1%)	
T2	22	(44%)	1	(5.9%)	21	(63.6%)		6	(21.4%)	16	(72.7%)	
T3	4	(8%)	0	(0%)	4	(12.1%)		0	(0%)	4	(18.2%)	

Lymph node												
Negative	36	(72%)	16	(94.1%)	20	(60.6%)	0.018^§^	27	(96.4%)	9	(40.9%)	<0.001^§^
Positive	14	(28%)	1	(5.9%)	13	(39.4%)		1	(3.6%)	13	(59.1%)	

NEDD9												
Mean ± SD	34.54	±24.15	8.64	±4.83	47.87	±18.49	<0.001^‡^	26.85	±24.43	44.31	±20.37	0.005^‡^
Median (range)	30	(3–90)	7	(3–20)	50	(20–90)		20	(3–90)	50	(5–80)	
Low	17	(34%)						14	(50%)	3	(13.6%)	0.007^§^
High	33	(66%)						14	(50%)	19	(86.4%)	

P38												
Mean ± SD	44.1 ± 28.1		30.2 ± 24.7		51.2 ± 27.5		0.016^‡^	20.5 ± 7.24		74.09 ± 10.07		<0.001^‡^
Median (range)	30	(10–90)	20	(10–90)	60	(10–90)		20	(10–35)	70	(60–90)	
Low	28	(56%)	14	(82.4%)	14	(42.4%)	0.007^§^					
High	22	(44%)	3	(17.6%)	19	(57.6%)						

Categorical variables were expressed as number (percentage); continuous variables were expressed as mean ± SD and median (range).

^‡^Mann–Whitney *U* test; ^§^Chi-square test; ^*∗*^Chi-square test for trend; *p* < 0.05 is significant.

**Table 3:** Association and correlation between NEDD9, P38,and clinicopathological parameters in 50 patients with TCC of bladder.

	NEDD9 (%)		NEDD9		P38 (%)		P38	
	*r*	*p* value	*r*	*p* value	*r*	*p* value	*r*	*p* value
Age	+0.152	0.293	−0.015	0.918	+0.448	0.001	+0.366	0.009
Sex	−0.392	0.005	−0.636	<0.001	−0.428	<0.001	−0.580	<0.001
Size	+0.601	<0.001	+0.366	0.009	+0.498	<0.001	+0.352	0.012
Multiplicity	+0.816	<0.001	+0.707	<0.001	+0.300	0.011	+0.356	0.007
Grade	+0.665	<0.001	+0.826	<0.001	+0.441	<0.001	+0.465	<0.001
Deep invasion	+0.630	<0.001	+0.552	<0.001	+0.535	<0.001	0.568	<0.001
T	+0.626	<0.001	+0.698	<0.001	+0.526	<0.001	+0.739	<0.001
Lymph node	+0.346	0.014	+0.354	0.012	+0.461	<0.001	+0.614	<0.001
NEDD9 (%)	—	—	—	—	+0.460	0.001	+0.362	0.010
NEDD9	—	—	—	—	+0.300	0.011	+0.381	0.007
P38 (%)	+0.460	0.001	+0.300	0.011	—	—	—	—
P38	+0.362	0.010	+0.381	0.007	—	—	—	—

*r* correlation coefficient; *p* < 0.05 is significant.

**Table 4:** Diagnostic performance of immunohistochemical markers as a predictor for deep invasion of TCC.

Markers	TP No (%)	FP No (%)	TN No (%)	FN No (%)	SN% (95% CI)	SP% (95% CI)	PPV% (95% CI)	NPV% (95% CI)	Accuracy (95% CI)
NEDD9	25 (50%)	8 (16%)	16 (32%)	1 (2%)	96.2% (88.8–100)	66.7% (47.8–85.5)	75.8% (61.1–90.4)	94.1% (82.9–100)	82% (71.4–92.6)
P38	20 (40%)	2 (4%)	22 (44%)	6 (12%)	76.9% (60.7–93.1)	91.7% (80.6–100)	90.9% (78.9–100)	78.6% (63.4–93.8)	84% (73.8–94.2)
NEDD9 & P38	19 (38%)	0 (0%)	27 (52%)	7 (14%)	73.1% (56–90.1)	100%	100%	79.4% (65.8–93)	86% (76.7–95.3)

TP: true positive; FP: false positive; TN: true negative; FN: false negative; SN: sensitivity; SP: specificity; PPV: positive predictive value; NPV: negative predictive value, 95% CI: 95% confidence interval; *p* < 0.05 is significant.

**Table 5:** Multivariate analyses for overall survival by Cox regression model.

Variable	Parameters	Hazard ratio	95% CI	*p* value
Age (years)	≤60 versus >60	0.83	0.322–2.175	0.7
Gender	Male versus female	0.34	0.129–0.972	0.006
Stage	≥T2 versus Ta-T1	9.8	2.25–42.88	<0.001
Grade	G3 versus G1/2	2.49	0.9–6.84	0.02
Number of tumors	Multiple versus single	0.49	0.3–1.09	0.02
Regional lymph node involvement	Present versus absent	0.02	0.0–4.795	<0.001
NEDD9	Low versus high	2.01	0.95–4.22	0.029
P38	Low versus high	3.7	1.81–7.94	<0.001

*p* < 0.05 is significant.
